# Chronic relapsing inflammatory optic neuropathy (CRION)

**DOI:** 10.1590/0004-282X-ANP-2022-E005

**Published:** 2022-06-24

**Affiliations:** 1 Harvard Medical School, Massachusetts General Hospital, Department of Neurology, Boston MA, USA.; 2 Harvard Medical School, Massachusetts Eye & Ear, Department of Ophthalmology, Boston MA, USA.

Optic neuritis (ON) is an inflammatory condition involving the optic nerve. In its most typical form, it is an acute unilateral disease characterized by vision loss and pain with eye movements, manifesting usually in young adults, especially females, between 18 and 45 years of age^1–3^. ON can be idiopathic or associated with recurrent demyelinating diseases, classically and most commonly, multiple sclerosis (MS) but also the more recently recognized antibody-associated diseases neuromyelitis optica spectrum disorder (NMOSD) and myelin oligodendrocyte glycoprotein antibody disease (MOGAD).

The clinical characteristics and natural history of typical ON were well characterized by the landmark Optic Neuritis Treatment Trial (ONTT)^1–3^. The ONTT demonstrated that the natural course of visual function after an episode of typical ON, either treated or untreated, is one of a rapid visual recovery within two weeks after onset of symptoms, with most recovery often taking place by four to six weeks and further slow recovery over several months, even up to one year. The ONTT conclusively demonstrated the strong association of ON with later development of MS. However, it also showed that ON can be recurrent, and that for reasons that remain somewhat unclear, in the ONTT ON recurrences occurred more commonly in patients treated with oral prednisolone alone. More specifically, within 2 years from diagnosis, the probability of recurrence in either eye was almost 2-fold higher in the prednisone group (30%) than in either the placebo group (14%) or the intravenous group (16%)^1–3^.

In the three decades since its completion, it has become clear that in many cases, ON does not behave in the typical fashion that was described and characterized in the ONTT. Atypical ON includes that associated with the antibody-associated demyelinating diseases NMOSD, which typically occurs in association with antibodies to aquaporin 4 as well as MOGAD, which is defined by antibody positivity to myelin oligodendrocyte glycoprotein (MOG). These diseases differ from typical and MS-associated ON in their clinical characteristics and natural history, including the propensity towards causing bilateral, longitudinally extensive and frequently more severe ON. For instance, the visual outcome after NMOSD ON events is less favorable compared to MS and MOGAD-related ON^4–6^. This is supported by greater thinning of RNFL (retinal nerve fiber layer) and GCL (ganglion cell layer) in NMOSD cases compared to typical ON^7^. However, MOGAD carries a propensity towards more frequent ON recurrence, and repeated episodes of ON in MOGAD patients often lead to comparable to NMOSD thinning in the OCT indices^8^. MOGAD-associated ON also appears to be highly steroid-responsive and, in some cases, steroid-dependent. In addition to the clinical course, efficient treatment of ON in NMOSD and MOGAD differs crucially from typical MS-related ON, making accurate diagnosis of these entities essential.

CRION (Chronic/relapsing inflammatory optic neuropathy) is emerging as a unique category of inflammatory ON that is characterized by it relapsing and/or chronic time course as well as its steroid responsiveness^9^. Orbital pain and/or pain with eye movements is frequently present. A defining characteristic of patients with CRION already noted in the original description^10^ is the rapid and excellent response to corticosteroid therapy but with a tendency to develop steroids dependence, with relapses within weeks or months after the withdrawal or decrease of corticosteroids. With accumulating clinical experience since then, it has become apparent that although CRION patients generally tend to initially respond well to steroids, cumulative damage can lead to poor visual outcomes and permanent loss of RNFL and GCL. Consequently, early diagnosis and timely management are key for restoring as well as preserving vision.

CRION was first described on clinical grounds in 2003, prior to the characterization of both aquaporin 4 and MOG antibodies^10^. It overlaps substantially with ON associated with MS, NMOSD and MOGAD, as all three of these disorders can be cause recurrent ON. MOGAD in particular more frequently presents with a CRION-like disease course than the other two demyelinating diseases^11^. However, a group of patients remains that is affected by chronic/relapsing ON without meeting criteria for MS, NMOSD or MOGAD, leading to the conclusion that CRION should be considered a distinct demyelinating disease. Importantly, CRION requires careful consideration and differentiation from typical, demyelinating ON, since the treatment is entirely different and the outcome without treatment is likely to be poor. Currently, no specific serologic biomarkers exist to help diagnose CRION, and radiologic biomarkers are likewise not specific. Therefore there is a need for better clinical understanding of this entity.

In the current edition of this journal, Molina-Carrión et al.^12^ contribute a report aimed at better characterizing the natural history of CRION. Out of a cohort of 1,735 patients with demyelinating disorders seen at a single tertiary referral center in Mexico, they identified 30 patients with CRION. In this study a high percentage (97%) of patients presented with ocular pain, and the great majority (87%) had bilateral involvement, though in most cases this was not present initially but rather developed sequentially. In most patients (77%), vision loss was severe (less than 20/100). Only one patient was found to have positive aquaporin 4 antibodies, although not all patients were tested and details of the serologic testing techniques were not provided. MOG antibody testing was institutionally unavailable. After acute management with steroids and sometimes plasmapheresis, patients were eventually treated with longer-term immune suppression with rituximab or mycophenolate mofetil, and the authors show that there is apparent benefit from this approach, as the annualized relapse rate decreased from 4.5 to 1.4 relapses per year after institution of longer-term immune suppression. In sum, within the limitations of a single-center and retrospective study, Molina-Carrión et al. are to be commended for contributing useful clinical characterization of CRION, a difficult and vexing, unique demyelinating disease with potential for chronic and severe visual morbidity.
